# Influence of *CYP2D6* and *CYP3A5* Polymorphisms on the Pharmacokinetics and Pharmacodynamics of Bisoprolol in Hypertensive Chinese Patients

**DOI:** 10.3389/fmed.2021.683498

**Published:** 2021-09-09

**Authors:** Sze Wa Chan, Tanya T. W. Chu, Chung Shun Ho, Alice P. S. Kong, Brian Tomlinson, Weiwei Zeng

**Affiliations:** ^1^School of Health Sciences, Caritas Institute of Higher Education, Hong Kong, China; ^2^Department of Medicine and Therapeutics, The Chinese University of Hong Kong, Prince of Wales Hospital, Hong Kong, China; ^3^Department of Chemical Pathology, The Chinese University of Hong Kong, Prince of Wales Hospital, Hong Kong, China; ^4^Faculty of Medicine, Macau University of Science and Technology, Macau, China; ^5^Shenzhen Baoan Women's and Children's Hospital, Jinan University, Shenzhen, China

**Keywords:** bisoprolol, blood pressure, *CYP2D6*, *CYP3A5*, pharmacokinetics, polymorphisms

## Abstract

**Purpose:** This study was performed to investigate the effects of common polymorphisms in *CYP2D6* and *CYP3A5* on the plasma concentrations and antihypertensive effects of bisoprolol in hypertensive Chinese patients.

**Methods:** One hundred patients with essential hypertension were treated with open-label bisoprolol 2.5 mg daily for 6 weeks. Clinic blood pressure (BP) and ambulatory BP (ABP) were measured after the placebo run-in and after 6 weeks treatment. Peak plasma concentrations of bisoprolol were measured at 3 h after the first dose and 3 h after the dose after 6 weeks treatment. Trough levels were measured before the dose after 6 weeks treatment. Bisoprolol plasma concentrations were measured with a validated liquid chromatography tandem mass spectrometry method. Six common polymorphisms in *CYP2D6* and the CYP3A5^*^3 polymorphism were genotyped by TaqMan® assay.

**Results:** After 6 weeks of treatment, clinic BP and heart rate were significantly reduced by 14.3 ± 10.9/8.4 ± 6.2 mmHg (*P* < 0.01) and 6.3 ± 7.6 BPM (*P* < 0.01), respectively. Similar reductions were seen in ABP values. Bisoprolol plasma concentration at 3 h after the first dose and 3 h post-dose after 6 weeks of treatment were significantly associated with baseline body weight (*P* < 0.001) but there was no significant effect of the *CYP2D6* and *CYP3A5* polymorphisms on these or the trough plasma concentrations. There was no significant association of the *CYP2D6* and *CYP3A5* polymorphisms or plasma bisoprolol concentrations with the clinic BP or ABP responses to bisoprolol.

**Conclusion:** Bisoprolol 2.5 mg daily effectively reduced BP and HR. The common polymorphisms in *CYP2D6* that were examined and the CYP3A5^*^3 polymorphism appear to have no benefit in predicting the hemodynamic response to bisoprolol in these patients.

## Introduction

Hypertension is the leading preventable cause of death globally, but there are major disparities of hypertension prevalence, awareness, treatment, and control in different countries (1). Beta-adrenoceptor antagonists or β-blockers are one of the oldest groups of drugs used to treat hypertension, although recent guidelines from the U.S. and Europe no longer recommend them as first line treatment for hypertension unless there are additional indications such as heart failure or post-myocardial infarction (2, 3). This advice is based on a meta-analysis which reported an increased risk of stroke with the use of β-blockers compared with other antihypertensive agents (4). However, in another meta-analysis which separated trials into those enrolling older patients ≥ 60 years and those enrolling younger patients <60 years, β-blockers demonstrated similar efficacy to other antihypertensive agents in younger patients but not in older patients (5). Hypertension Canada's 2020 Guidelines still recommends that β-blockers may be used as first-line monotherapy in patients younger than 60 years of age but not in patients aged ≥ 60 years (6).

The blood pressure responses to β-blockers and other antihypertensive drug classes is partly dependent on genetic variation in both pharmacodynamic and pharmacokinetic pathways (7). Several β-blockers, in particular metoprolol, are extensively metabolized by cytochrome P450 2D6 (*CYP2D6*) and the *CYP2D6* genotype has a pronounced effect on the single dose pharmacokinetics of metoprolol which persists during long-term therapy (8). The effect of *CYP2D6* genotype on the hemodynamic and clinical responses to metoprolol has not been consistent but a meta-analysis of seven studies in 2017 concluded that *CYP2D6* polymorphisms significantly influenced the heart rate (HR) and diastolic blood pressure (DBP), but not the systolic blood pressure (SBP) response to metoprolol (9). A more recent meta-analysis of 15 studies found that *CYP2D6* poor metabolizers (PM) had significantly greater reductions in HR, SBP and DBP compared to non-PM individuals (10).

Bisoprolol is a moderately lipophilic highly β_1_-selective β-blocker that is devoid of any intrinsic sympathomimetic activity (ISA), vasodilatory effects or membrane stabilizing properties. It is one of the few β-blockers approved for congestive heart failure in addition to the usual β-blocker indications of hypertension and coronary heart disease (11). Bisoprolol is eliminated with 50% renal excretion as unchanged drug and 50% *via* hepatic metabolism to pharmacologically inactive metabolites which are then excreted by the kidneys (11). It is reported to be metabolized by *CYP3A4* and to a small extent by *CYP2D6* (12, 13).

*CYP3A4* is abundantly expressed in human liver and intestine, representing 30–50% and 70% of the two microsomal P450 pools, respectively (14). *CYP3A5*, which is expressed in intestinal enterocytes and in other extra-hepatic tissues, may contribute up to about 50% of the *CYP3A* pool in individuals with low *CYP3A4* expression (14, 15). The CYP3A5^*^3 (rs776746, 6986G>A) polymorphism is a common variant occurring in all populations with an allele frequency of 0.65 in Chinese (16).

*CYP2D6* is highly polymorphic with 145 variant alleles reported so far, many of these having reduced or absent function (17). CYP2D6^*^1, ^*^2, ^*^5, and ^*^10 were the most frequent *CYP2D6* alleles found in Hong Kong Chinese (18, 19). The reduced-function CYP2D6^*^10 allele is the most common variant in East Asians and occurs in 33–43% of these populations, including Japanese, Korean, and Chinese, but in only about 2–5% in Caucasians and African Americans (20). Conversely, the frequency of the loss of gene variant (CYP2D6^*^5) is similar among different ethnic groups (4–7%) (21). The CYP2D6^*^14B allele, which differs from the ^*^14 allele by the absence of the C100T substitution and by the additional G1749C substitution, occurs in 2% of Chinese (22). Nozawa et al. (23) reported an association between *CYP2D6* polymorphisms and plasma concentrations of metoprolol but not bisoprolol. Scoring systems have been established in an attempt to provide *CYP2D6* alleles a uniform approach to quantitate the predicted functional status (24) and these have been updated recently (17). Poor metabolizers (PMs) differ from extensive metabolizers (EMs) by 5- to 15-fold if determined by rates of metabolism or by ratios of parent to metabolite concentrations (25, 26).

The influence of the CYP3A5^*^3 polymorphism in the overall oxidative activity of CYP3A and the possible relation of CYP3A5^*^3 and CYP3A4^*^1G polymorphisms on CYP3A activity and their potential interaction is still uncertain (27, 28). In addition, the influence of *CYP2D6* genotypes on the pharmacokinetics and therapeutic responses of bisoprolol have been inconsistent (29). The present study, therefore, investigated the effect of *CYP3A5* and *CYP2D6* genotypes on the plasma concentrations of bisoprolol and the clinic and 24-h ambulatory blood pressure (ABP) responses in Chinese patients with mild to moderate hypertension.

## Materials and Methods

### Study Participants

A total of 141 patients with a *de novo* diagnosis of primary hypertension or a previous history of primary hypertension identified from outpatient clinics in the Prince of Wales Hospital, Hong Kong were invited to participate. Sitting clinic BP levels after a placebo run-in of at least 2 weeks were required to be in the range of SBP 140–169 mmHg and/or DBP of 90–109 mmHg in otherwise healthy patients or SBP 130–169 mmHg and/or DBP of 80–109 mmHg in patients with diabetes mellitus. After informed consent was obtained, subjects were withdrawn from any previous antihypertensive therapy and given placebo once daily for at least 2 weeks. Amlodipine treatment was continued if necessary to achieve BPs in the defined range at the end of the placebo run-in. Compliance was assessed using pill counting, and any subject with compliance <80% during the placebo run-in period was excluded from the study.

Individuals with secondary hypertension, unstable angina, a history of myocardial infarction, stroke or coronary heart disease (coronary by-pass or angioplasty) in the previous 3 months before recruitment, heart failure (New York Heart Association [NYHA] II–IV), hemodynamically relevant aortic or mitral valve disease, hypertrophic obstructive cardiomyopathy, symptomatic bradycardia, second or third degree AV block, sick sinus syndrome, sinoatrial block, or HR <70 beats/min (BPM) at baseline (before starting bisoprolol treatment), primary hyperaldosteronism, renal artery stenosis, impairment of hepatic or renal function as defined by liver function values of ALT ≥ 1.5-fold the upper limit of normal or serum creatinine >150 μmol/L or upon investigator decision, and history or intolerance or with a known contraindication to β-blockers were excluded.

### Study Design

This was a phase IV clinical trial registered with reference number NCT02398929 (https://clinicaltrials.gov/show/NCT02398929). Patients were enrolled into an open-label, pharmacogenetic study of bisoprolol treatment with a placebo run-in of at least 2 weeks. According to the total duration of bisoprolol treatment, the study participants were divided into two groups. In group A, 63 patients were screened and 50 were enrolled and given bisoprolol 2.5 mg once daily for 6 weeks. They had venous blood samples collected after 6 weeks of treatment before the dose for trough drug concentration assay. In group B, 78 patients were screened and 50 were enrolled and were treated with bisoprolol 2.5 mg once daily for 6 weeks and then continued treatment for a total of 24 weeks with optional titration of the dose of bisoprolol by doubling the dose after 6-week intervals up to 10 mg to achieve target BP levels. In this group, additional venous blood samples were collected 3 h after the first dose and 3 h after the dose after 6 weeks of treatment for peak plasma concentrations of bisoprolol. Clinic BP and 24-h ABP measurements were made at baseline, after the first dose of bisoprolol 2.5 mg and at the end of 6 weeks treatment with bisoprolol 2.5 mg. The patients were instructed to wear a wrist-type (BPro, HealthSTATS International, Singapore) or arm-type (A&D TM-2430, Tokyo, Japan) ABP device for 24 h and their BPs were measured at intervals automatically throughout 24 h. Some patients were fitted with both ABP devices to compare the readings. The wrist monitor showed reasonable agreement with the arm monitor in previous studies (30). Patients were encouraged to continue their usual daily activities but not to engage in vigorous physical exercise such as running, climbing, or playing sports. A daily activity record form was given to each patient.

### Ethics

The study involving human participants was reviewed and approved by the Joint Clinical Research Ethics Committee of the Chinese University of Hong Kong and New Territories East Cluster (CUHK-NTEC) with reference number CRE-2011.616-T. The study was performed in accordance with the ethical standards laid down in the Declaration of Helsinki and subsequent revisions. All patients signed the Informed Consent.

### Biochemical Assessments

Plasma lipid profile (total cholesterol, triglycerides, and HDL-cholesterol), glucose, renal, and liver function tests were measured on a Roche Modular Analytics system (Roche Diagnostics GmbH, Mannheim, Germany) using standard reagent kits supplied by the manufacturer of the analyzer. The analytical performance of these assays was within the manufacturer's specifications. Low-density lipoprotein cholesterol level was estimated by using the Friedewald formula (31) or directly measured when the TG level was over 4.5 mmol/L.

Glycosylated hemoglobin (HbA1c) was measured using an automated ion-exchange chromatographic method (Bio-Rad Laboratory, Hercules, CA; reference range 5.1–6.4%). The inter-assay and intra-assay coefficient of variation (CV) for HbA1c was 3.1% at values <6.5%.

### Genotyping

Six common polymorphisms in *CYP2D6* [^*^10 (100C>T, rs1065852), ^*^4 (1934G>A, rs3892097, 1846G>A/T, rs5030865), ^*^2 (2938C>T, rs16947, 4268G>C, rs1135840) and ^*^5, deletion] and the CYP3A5^*^3 (rs776746, 6986G>A) polymorphism were selected in this study. DNA was extracted from peripheral whole blood samples by the phenol chloroform method. Genetic polymorphisms in *CYP2D6* and the CYP3A5^*^3 polymorphism were genotyped by TaqMan® assay using the geneAmp PCR system 9700 (Applied Biosystems, Foster City, CA, USA). The CYP3A5^*^3 polymorphism was determined using a previously reported polymerase chain reaction (PCR) restriction fragment length polymorphism (RFLP) method (32), while a long-PCR technology was used to detect the CYP2D6^*^5 variant as described previously (33). The 5 kb of the *CYP2D6* gene was amplified first and then diluted 100-fold with water before performing the Taqman assay. The Taqman assays and detection were performed with ViiA7 from Life Technologies. The specific pair of primers used for PCR was as follows: Forward primer, 5′-CCA GAA GGC TTT GCA GGC TTC A-3′, and reverse primer, 5′-ACT GAG CCC TGG GAG GTA GGT A-3′. The PCR reaction conditions for *CYP2D6* were: initial denaturation at 94°C for 2 min, followed by 10 cycles of denaturation at 94°C for 1 min, annealing at 60°C for 30 s, extension at 68°C for 4 min, and followed by 30 cycles of denaturation at 94°C for 1 min, annealing at 60°C for 30 s, extension at 68°C for 4 min and 20 s, and the final extension at 68°C for 7 min.

All polymorphisms examined in this study were in Hardy–Weinberg equilibrium (χ^2^ test *P* > 0.05) and the frequencies of the minor alleles were similar to those reported in Han Chinese in HapMap. For translation of the genotypes into a qualitative measure of metabolizer group, the *CYP2D6* activity score of each subject was calculated as the sum of the values assigned to each single allele (17). Subjects with an activity score of 1.25–2.25 were classified as normal metabolizers (NMs) whereas subjects with a score of 0 were classified as poor metabolizers (PMs) and subjects with a score of <1.25 was classified as intermediate metabolizers (IMs); subjects with a score > 2.25 were to be classified as ultra-rapid metabolizers (UMs) but none were identified.

### Bisoprolol Assay

Plasma concentrations of bisoprolol were determined with a validated bioanalytical method. Method development and validation was performed according to the U.S. Food and Drug Administration (USFDA) guidance on Bioanalytical Method Validation (34). Briefly, liquid-liquid extraction was used to extract analyte and deuterium-labeled internal standard, bisoprolol-D7, from the biological matrix. After extraction, the target compounds were separated on a Waters ACQUITY BEH C18 UPLC column (2.1 × 50 mm, 1.7 μm), from 55% mobile phase A (0.1% formic acid in MilliQ water) to 80% mobile phase B (100% HPLC grade methanol) in 2 min, followed by 1 min washing at 95% mobile phase B and 1 min re-equilibration and detected by electrospray ionization tandem mass spectrometry using multiple reaction monitoring (MRM) in positive ion mode. Bisoprolol and the internal standard were both eluted at around 1 min and monitored by MRM transition m/z 326 > 116 and m/z 333 > 123, respectively. MRM transition 326 > 98 was used for bisoprolol as qualifier. Pooled plasma was spiked with a working solution to give 34, 1.7, and 0.34 μg/L quality control (QC) samples. An extra level of 0.1 μg/L was prepared for the lower limit of quantitation (LLOQ) validation purposes. All QC samples were aliquoted and stored at −80°C. This method covered a concentration range from 0.1 to 81.5 μg/L and total imprecision was <6% (<4% when excluding LLOQ) while inaccuracy was <13% throughout the concentration range (<4% when excluding LLOQ), which were within the acceptance criteria of Food and Drug Administration (FDA) and National Medical Products Administration (NMPA) guidelines. The imprecision and inaccuracy of LLOQ was 5.2 and −12.4%, respectively, both of which were better than the requirement by FDA and CFDA (+20%). Recovery and process efficiency of the assay at different concentrations were both on average 89%, suggesting a minimal loss of analyte during sample preparation.

### Statistical Analysis

Statistical analyses were performed using IBM SPSS software (Version 26, IBM SPSS Inc., Armonk, New York, USA). Data were pooled from the two groups of subjects. The distribution of continuous data was evaluated according to the Shapiro–Wilk test. Differences in baseline characteristics, blood pressure and lipid profiles between the two studies were assessed using Student's *t*-test or Mann–Whitney *U*-test, as appropriate. χ^2^ test were used to test Hardy–Weinberg equilibrium and comparisons for categorical variables. Logistic regression analyses were applied to determine significantly independent predictors of BP and HR response and the pharmacogenetic analysis. Paired Student's *t*-test was used to compare the peak plasma levels of bisoprolol concentration 3 h post-first dose and 3 h post-dose after 6 weeks of treatment. The bisoprolol plasma concentrations were adjusted for body weight based on the univariate analysis. An independent samples *t*-test or an analysis of covariance (ANCOVA) followed by Tukey's multiple comparison test was used to assess the effect of the genetic polymorphisms on plasma concentrations of bisoprolol with body weight as covariate. Statistical analysis on the effect of genetic polymorphisms on the BP and HR responses to bisoprolol was performed using an independent samples *t*-test or a one-way analysis of variance (ANOVA) followed by Tukey's multiple comparison test, as appropriate. Data are presented as mean ± standard deviation unless otherwise specified. A *P* < 0.05 was considered statistically significant.

## Results

### Study Population

**Figure 1:**
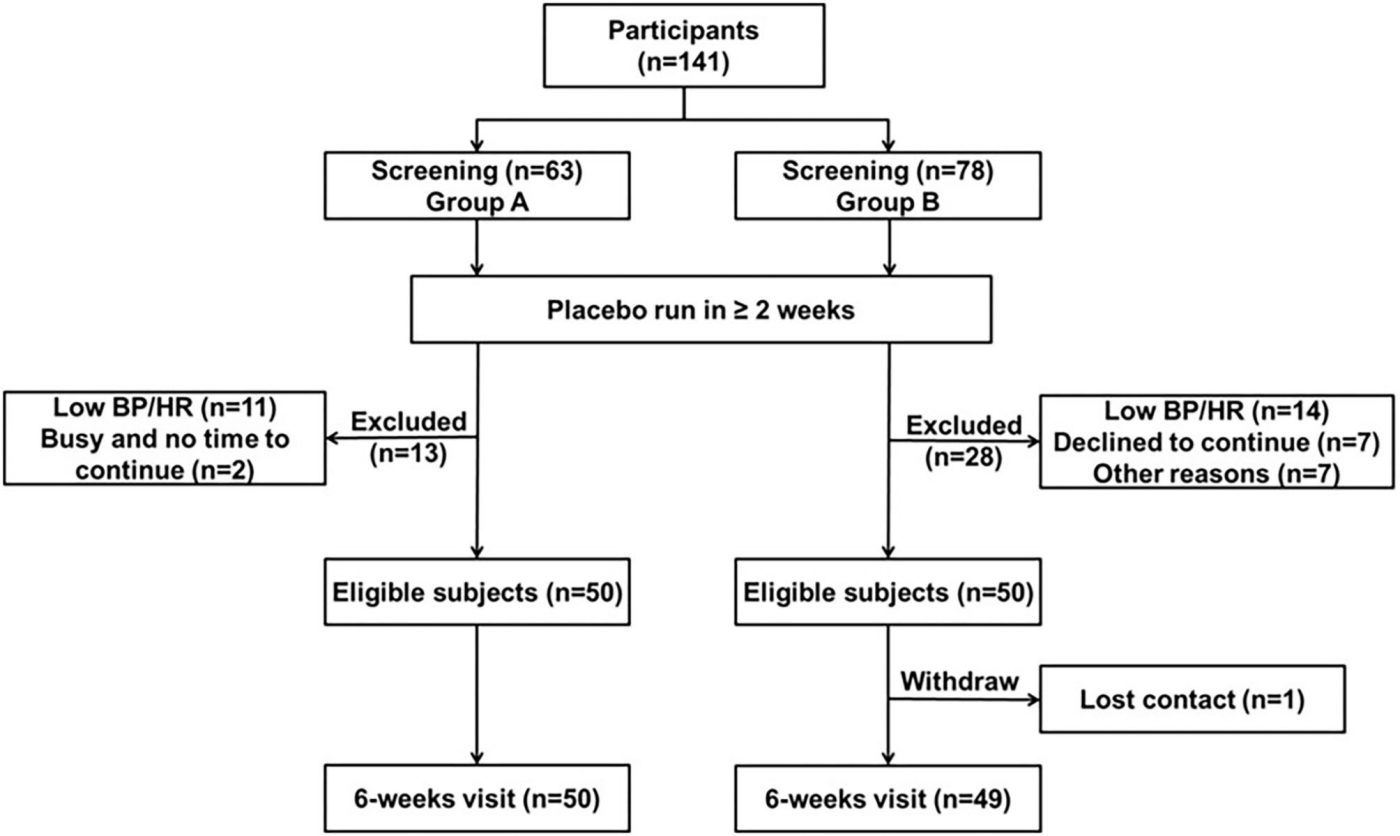
Consort diagram of the study.

Fifty patients completed 6 weeks of bisoprolol treatment in group A and in group B, 49 patients completed the first 6 weeks treatment with bisoprolol and seven of them withdrew from the study subsequently (see Figure 1). The demographic and baseline characteristics and concomitant diseases of the study patients are shown in Table 1. All patients were of Chinese ethnicity (99 subjects), with mean (± SD) age 54 ± 10 years. The median body weight was 66.2 kg (25th−75th percentiles 57.5–77 kg) and median BMI 25.1 kg/m^2^ (22.7–27.9 kg/m^2^). The mean baseline clinic BP was 144.1 ± 10.6 mmHg/ 92.2 ± 9.3 mmHg. Patients had relatively normal lipid and glycemic profiles, as most of them were under medication control. There were no significant differences in the BPs, lipid profile, and glycemic profile between the two study groups, except the HbA1c level as there were more patients with diabetes in group B. The subjects were required to stop all the anti-hypertensive medication except amlodipine, of which 21 subjects were taking at a constant dose together with bisoprolol during the study.

**Table 1:** Characteristics of the study population.

**Parameters**	**Total**	**Group A**	**Group B**	***P* value**
*N* (% male)	99 (60%)	50 (62%)	49 (56%)	0.627
Age (years)	54 ± 10	53 ± 10	55 ± 11	0.566
BMI (kg/m^2^)	25.1 (22.7–27.9)	23.8 (22.4–27.2)	25.4 (23.0–28.3)	0.139
Body weight (kg)	66.2 (57.5–77.0)	64.9 (57.4–75.5)	68.9 (60.6–77.7)	0.389
Current smoker	8 (8%)	4 (8%)	4 (8%)	1.000
Drinker	8 (8%)	5 (10%)	3 (6%)	0.469
Creatinine (μmol/l)	76.0 (61.0–87.0)	76.0 (64–91)	74.0 (58–82)	0.131
eGFR (ml/min/1.73 m^2^)	11.5 (99.6–128.7)	108.7 (94.2–121.5)	118.2 (100.9–130.3)	0.067
TC (mmol/l)	4.9 ± 0.8	4.9 ± 0.7	4.9 ± 0.8	0.777
HDL-C (mmol/l)	1.3 (1.1–1.6)	1.40 (1.10–1.60)	1.20 (1.03–1.50)	0.211
LDL-C (mmol/l)	2.85 (2.30–3.30)	2.90 (2.50–3.23)	2.80 (2.30–3.30)	0.584
TG (mmol/l)	1.6 ± 0.7	1.5 ± 0.7	1.7 ± 0.8	0.199
nHDL (mmol/l)	3.6 ± 0.8	3.6 ± 0.8	3.6 ± 0.8	0.945
FPG (mmol/l)	5.1 (4.7–5.6)	5.1 (4.8–5.5)	5.2 (4.7–6.2)	0.295
HbA1c (%)	5.6 (5.3–6.0)	5.3 (5.1–5.6)	5.9 (5.6–6.3)	<0.0001
Clinic SBP (mmHg)	144.1 ± 10.6	146.2 ± 10.4	141.8 ± 10.4	0.039
Clinic DBP (mmHg)	92.2 ± 9.3	92.4 ± 10.4	91.9 ± 8.1	0.793
Clinic HR (beats/min)	71.6 ± 10.4	71.8 ± 11.4	71.4 ± 9.5	0.880
Ambulatory SBP (mmHg)	143.1 ± 11.3	143.0 ± 11.7	143.1 ± 11.0	0.966
Ambulatory DBP (mmHg)	92.1 ± 9.1	90.6 ± 9.3	93.6 ± 8.8	0.103
Ambulatory HR (beats/min)	74.8 ± 7.4	74.9 ± 8.4	74.8 ± 6.2	0.985
Daytime SBP (mmHg)	147.1 ± 11.5	147.2 ± 12.1	147.0 ± 11.1	0.910
Daytime DBP (mmHg)	94.8 ± 9.2	93.5 ± 9.4	96.0 ± 9.0	0.182
Daytime HR (beats/min)	77.9 ± 8.0	77.6 ± 9.1	78.1 ± 6.6	0.771
Nighttime SBP (mmHg)	132.7 ± 14.6	130.8 ± 16.0	134.6 ± 12.9	0.203
Nighttime DBP (mmHg)	84.3 ± 11.7	81.3 ± 10.5	87.5 ± 12.1	0.008
Nighttime HR (beats/min)	66.6 ± 8.0	65.2 ± 8.3	68.0 ± 7.4	0.082
**Medical history**
Hyperlipidemia (*n*)	41	19	23	
Diabetes (*n*)	13	5	8	
**Medication record**
With amlodipine (*n*)	21	0	21	
Without amlodipine (*n*)	78	50	28	

*Data are expressed as mean ± SD or median (25th and 75th Percentile). Differences between the two groups were compared by Student's t-test or Mann–Whitney U-test, as appropriate*.

*BMI, body mass index; eGFR, estimated glomerular filtration rate; TC, total cholesterol; HDL-C, high-density lipoprotein cholesterol; TG, triglyceride; LDC-C, low-density lipoprotein cholesterol; nHDL, non-high-density lipoprotein cholesterol; FPG, fasting plasma glucose; HbA1c, glycosylated hemoglobin; SBP, systolic blood pressure; DBP, diastolic blood pressure; HR, heart rate*.

Demographic data of study participants according to *CYP2D6* and *CYP3A5* genotypes are shown in Table 2. The observed *CYP2D6* allele frequencies were 29% for ^*^1, 11% for ^*^2, 2% for ^*^14B, 55% for ^*^10, and 3% for ^*^5. No patients carried the UM genotype in the present study. The distributions of *CYP2D6* genotypes, metabolizer groups and *CYP3A5* genotypes are shown in Table 2. There were two patients with a very high bisoprolol concentrations (18.5 μg/l before dose and 40.1 μg/l at 3 h post dose after 6 weeks of treatment) and they were considered as outliers possibly due to experimental error and these values were excluded from the analysis. Thus, we combined the data from 97 patients who finished 6 weeks of bisoprolol treatment in each of the two studies.

**Table 2:** Demographic data of study subjects (*n* = 99) according to *CYP2D6* metabolizer and *CYP3A5* genotype groups.

**Genotypes**	**Genotype score (*N*)**	**Metabolizer group**	**No. of subjects**	**Age**	**Sex (% male)**	**Body weight (kg)**	**Change in clinic SBP**	**Change in clinic DBP**	**Change in clinic HR**	**Bisoprolol** **concentration 3 h post-first dose (μg/mL)**	**Bisoprolol** **concentration** **before dose after 6 weeks (μg/mL)**	**Bisoprolol** **concentration** ** h post-dose after 6 weeks (μg/mL)**
***CYP2D6***											
*1/*2	2 (11)	NM	56	53.8 ± 10.2	59%	66.2 (58.1–75.2)	14.7 ± 8.8	8.9 ± 4.8	6.0 ± 5.2	13.5 ± 2.7	3.3 ± 2.1	15.3 ± 3.4
*2/*2	2 (1)											
*1/*1	2 (9)											
*1/*10	1.25 (27)											
*2/*10	1.25 (8)											
*2/*14B	1 (1)	IM	42	51.0 ± 9.0	83%	62.0 (59.9–87.5)	12.5 ± 5.5	7.5 ± 2.9	8.5 ± 4.0	13.4 ± 2.8	2.7 ± 1.1	13.2 ± 2.6
*1/*14B	1 (1)											
*10/*10	0.5 (34)											
*5/*10	0.25 (5)											
*14B/*10	0.25 (1)											
*5/*14B	0 (1)	PM	1	65	0%	49	35	7	7	16.34	5.62	19.1
***CYP3A5***											
*1/*1		NM	46	54.9 ± 10.2	54%	65.5 (56.8–77.0)	13.4 ± 8.1	7.6 ± 4.5	6.8 ± 6.4	14.0 ± 2.4	3.2 ± 1.5	15.4 ± 3.4
*1/*3		IM	43	52.4 ± 10.6	65%	68.9 (62.2–77.2)	15.4 ± 8.1	9.6 ± 5.5	5.6 ± 5.5	12.6 ± 3.0	3.0 ± 2.2	14.6 ± 4.4
*3/*3		PM	10	56.3 ± 8.8	60%	64.7 (58.5–68.2)	13.3 ± 11.3	6.5 ± 3.8	7.0 ± 4.4	13.6 ± 2.4	2.8 ± 1.5	15.7 ± 4.2
All subjects			99	54 ± 10	60%	66.2 (57.5–77.0)	14.2 ± 8.5	8.4 ± 5.0	6.3 ± 5.8	13.3 ± 2.9	3.1 ± 1.9	15.0 ± 4.0

*Data are expressed as mean ± SD or median (25th percentile and 75th percentile)*.

*NM, normal metabolizer; IM, intermediate metabolizer; PM, poor metabolizer; SBP, systolic blood pressure; DBP, diastolic blood pressure; HR, heart rate*.

### Pharmacogenetic Analysis of Bisoprolol Plasma Concentrations

**Figure 2:**
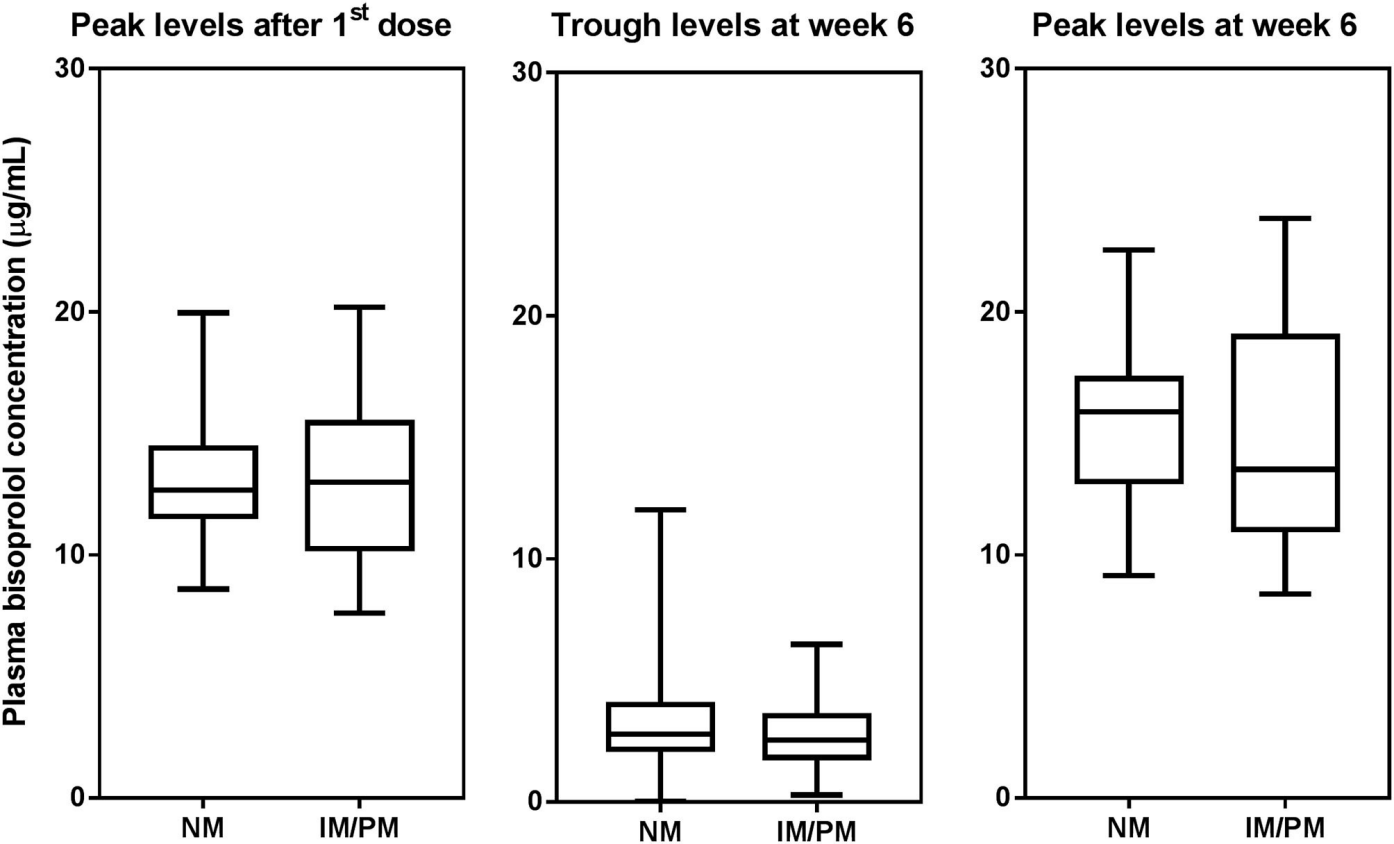
Box-and-whisker plot of plasma bisoprolol concentrations according to *CYP2D6* metabilizer groups. The boxes represent the 25th−75th percentiles, the whiskers represent the range. There were no significant differences between metabolizer groups by independent samples *t*-test.

**Figure 3:**
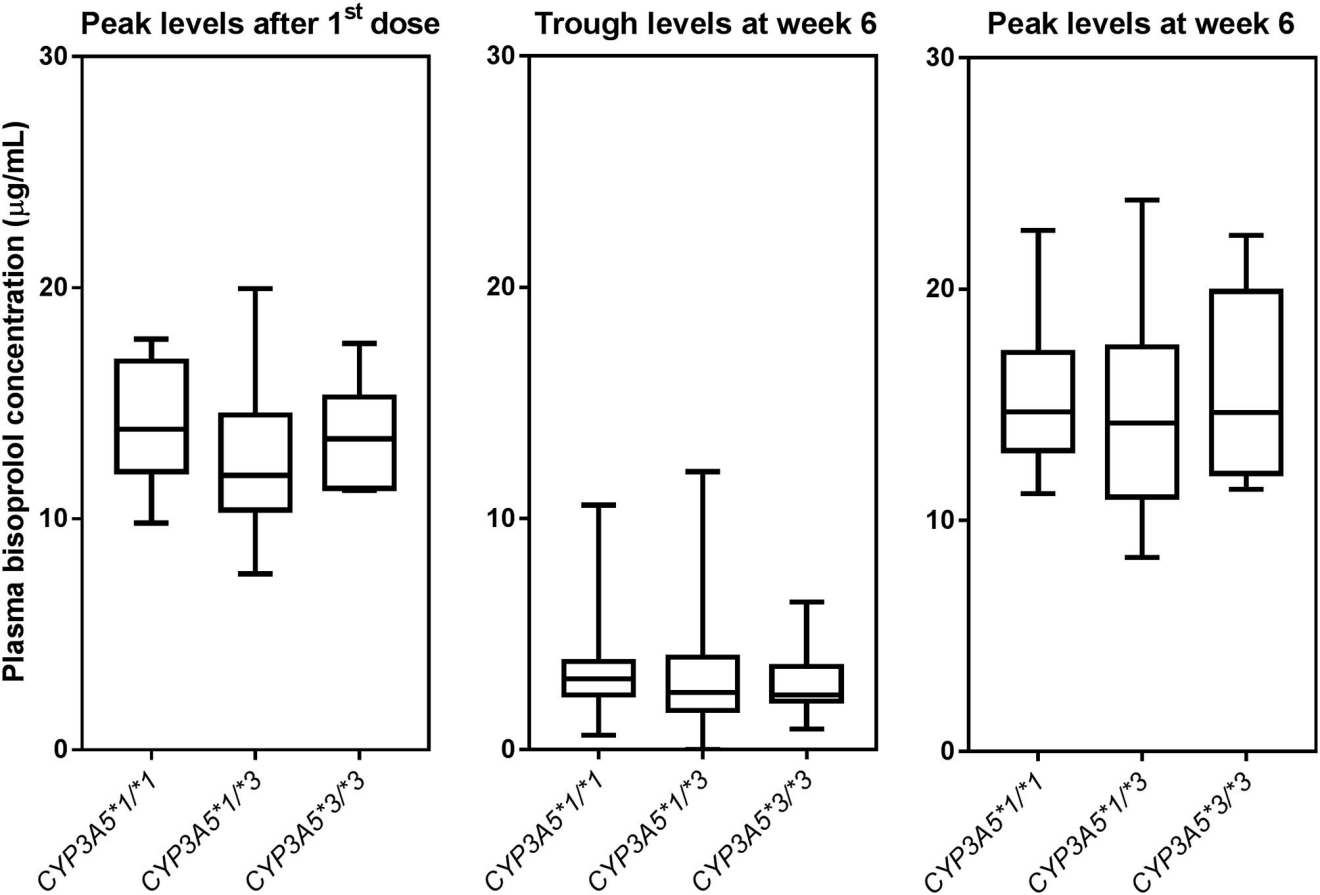
Box-and-whisker plot of plasma bisoprolol concentrations according to *CYP3A5* genotypes. The boxes represent the 25th−75th percentiles, the whiskers represent the range. There were no significant differences between genotype groups by one-way ANOVA, with and without adjustment for body weight.

Peak plasma concentrations of bisoprolol at 3 h after the first dose and 3 h after the dose after 6 weeks treatment were measured in 45 and 44 patients, respectively. Trough levels before the dose after 6 weeks treatment were determined in all 97 subjects. The peak levels after 6 weeks of treatment were increased by 13.6 ± 16.9% compared to the peak levels for first dose (peak level for first dose 13.2 ± 2.8, peak level at week 6 15.0 ± 4.0 μg/mL, *P* < 0.001). Univariate analysis showed that the bisoprolol concentration 3 h post first dose and 3 h post dose after 6 weeks of treatment was significantly related to body weight (*p* < 0.001) while there were no significant effects for other factors including sex, age, eGFR, concomitant treatment with amlodipine, or *CYP3A5* and *CYP2D6* genotype (Table 3). Only one subject had a *CYP2D6* genotype score of 0 and this subject was combined with the IM group in the analysis. Multiple linear regression analyses were carried out to test the association between those candidate predictors and bisoprolol concentration (Table 3). Body weight influenced the bisoprolol concentration at 3 h post first dose (*p* < 0.001) and 3 h post dose (*p* < 0.001) after 6 weeks stable treatment, while gender and *CYP2D6* metabolizer group and CYP3A5^*^3 genotype did not have any effect on peak bisoprolol concentrations in this study (Figures 2, 3). Moreover, a higher body weight predicted a lower peak plasma bisoprolol concentration. On the other hand, body weight and age were predictors of the trough bisoprolol concentration after 6 weeks treatment on univariate but not on multiple linear regression analysis (Table 3). There was no effect of *CYP2D6* metabolizer group and CYP3A5^*^3 genotype on trough bisoprolol concentration (Figures 2, 3).

**Table 3:** Linear regression analysis for factors that may influence bisoprolol peak and trough plasma concentrations.

	**Univariate**		**Multiple linear regression**	
	**B (95% CI for B)**	***P***	**B (95% CI for B)**	***P***
**Bisoprolol concentration 3 h post-first dose (** ***n*** **=** **45)**				
Sex	−3.970 (−5.146 to −2.794)	<0.001	−1.935 (−3.134 to −0.735)	0.002
Age	0.118 (0.046 to 0.190)	0.002	−0.002 (−0.056 to 0.052)	0.934
Body weight	−0.150 (−0.180 to −0.120)	<0.001	−0.118 (−0.161 to −0.074)	<0.001
*CYP2D6* genotype score	0.179 (−1.565 to 1.922)	0.837	0.562 (−0.356 to 1.481)	0.223
CYP3A5*3 genotype	−0.488 (−1.774 to 0.798)	0.448	−0.240 (−0.894 to 0.414)	0.462
eGFR	0.004 (−0.032 to 0.041)	0.821	−0.005 (−0.029 to 0.018)	0.641
Concomitant with amlodipine	−1.565 (−3.208 to 0.078)	0.61	0.393 (−0.552 to 1.338)	0.405
**Bisoprolol concentration 3 h post-dose after 6 weeks (** ***n*** **=** **44)**				
Sex	−4.828 (−6.755 to −2.901)	<0.001	−1.994 (−4.294 to 0.305)	0.087
Age	0.130 (0.022 to 0.239)	0.019	−0.039 (−0.143 to 0.065)	0.451
Body weight	−0.192 (−0.244 to −0.141)	<0.001	−0.164 (−0.246 to 0.083)	<0.001
*CYP2D6* genotype score	0.333 (−2.212 to 2.878)	0.793	0.563 (−1.241 to 2.367)	0.531
CYP3A5*3 genotype	−0.062 (−1.912 to 1.788)	0.946	0.192 (−1.047 to 1.432)	0.755
eGFR	0.008 (−0.045 to 0.060)	0.770	−0.010 (−0.054 to 0.035)	0.658
Concomitant with amlodipine	−2.749 (−5.080 to −0.418)	0.022	−0.350 (−2.176 to 1.476)	0.700
**Bisoprolol concentration before dose after 6 weeks (** ***n*** **=** **97)**				
Sex	−0.902 (−1.646 to −0.158)	0.018	−0.660 (−1.534 to 0.215)	0.137
Age	0.049 (0.013 to 0.085)	0.008	0.021 (−0.021 to 0.062)	0.325
Body weight	−0.051 (−0.076 to −0.025)	<0.001	−0.031 (−0.065 to 0.003)	0.077
*CYP2D6* genotype score	0.590 (−0.245 to 1.425)	0.164	0.502 (−0.302 to 1.306)	0.218
CYP3A5*3 genotype	−0.221 (−0.791 to 0.350)	0.445	−0.036 (−0.584 to 0.512)	0.896
eGFR	−0.015 (−0.032 to 0.002)	0.080	−0.013 (−0.032 to 0.006)	0.166
Concomitant with amlodipine	−0.659 (−1.582 to 0.263)	0.159	−0.025 (−0.956 to 0.906)	0.958

*eGFR, estimated glomerular filtration rate; CI, Confidence Interval*.

### Effect of *CYP2D6* and *CYP3A5* Genotypes on the BP and HR Response to Bisoprolol

**Figure 4:**
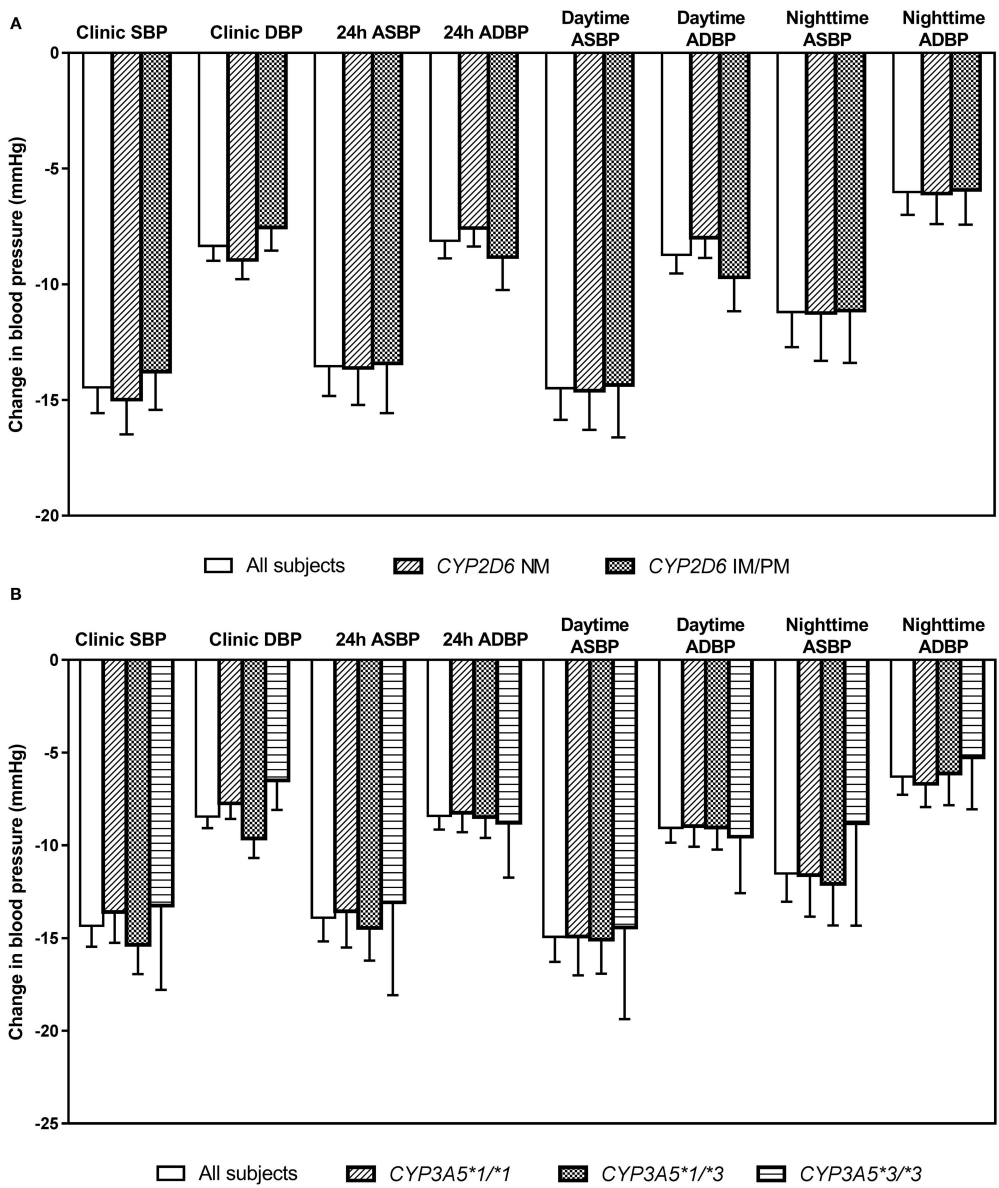
Changes in clinic and ambulatory blood pressure after 6 weeks treatment with bisoprolol 2.5 mg daily according to **(A)** *CYP2D6* metabolizer groups, and **(B)** *CYP3A5* genotypes. Data are presented as mean ± SEM. There is no significant difference between groups (independent samples *t*-test or one-way ANOVA, as appropriate).

**Figure 5:**
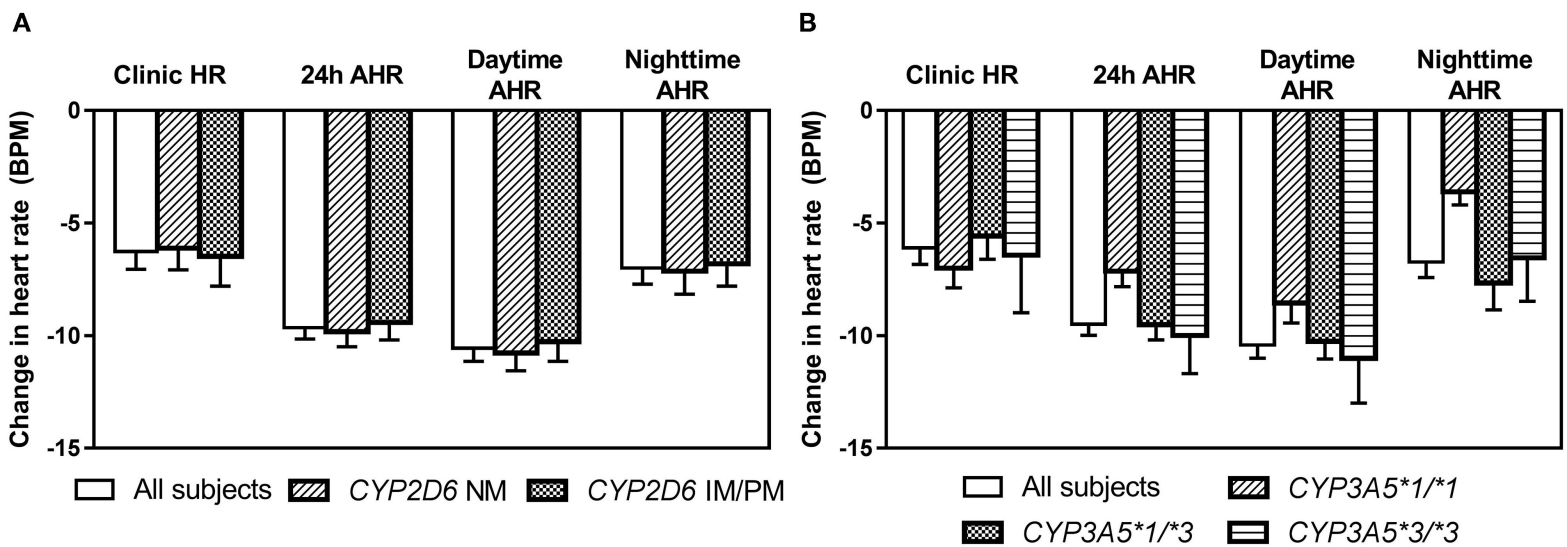
Changes in clinic heart rate and ambulatory heart rate (AHR) after 6 weeks treatment with bisoprolol 2.5 mg daily according to **(A)** *CYP2D6* metabolizer groups **(B)** *CYP3A5* genotypes. Data are presented as mean ± SEM. There is not significant difference between groups (independent samples *t*-test or one-way ANOVA, as appropriate).

After 6 weeks of treatment with bisoprolol 2.5 mg daily, reductions in clinic BP and HR were 14.3 ± 10.9/8.4 ± 6.2 mmHg (*P* < 0.01) and 6.3 ± 7.6 BPM (*P* < 0.01), respectively, and there were similar reductions in the ABP and HR values (data not shown). Univariate analysis showed that BP and HR responses to bisoprolol were significantly related to baseline BP and HR (Table 4). The bisoprolol concentration before dose after 6 weeks of treatment was not related to changes in BP and HR but the peak level at week 6 was significantly associate with changes in SBP (*P* < 0.01*)* and DBP (*P* < 0.01) but not HR. There was no significant effect for sex, age, body weight, and concomitant treatment with amlodipine (Table 4). The subject with a *CYP2D6* genotype score of 0 was combined with the IM group in the analysis and this subject actually had a small increase in BP with bisoprolol treatment. There was no significant difference in the clinical and ambulatory BP reductions among *CYP2D6* metabolizer groups and *CYP3A5* genotypes (Figures 4A,B). Similarly, no significant difference was observed in the clinic and ambulatory HR according to *CYP2D6* metabolizer groups and *CYP3A5* genotypes (Figures 5A,B).

**Table 4:** Linear regression analysis for the factors that may influence BP and HR reductions after 6 weeks of treatment.

	**Univariate**		**Multiple linear regression**	
	**B (95% CI for B)**	***P***	**B (95% CI for B)**	***P***
**Change in clinic SBP**				
Sex	4.518 (0.077 to 8.958)	0.046	0.100 (−4.572 to 4.771)	0.966
Age	−0.016 (−0.237 to 0.204)	0.883	0.093 (−0.125 to 0.312)	0.398
Body weight	0.191 (0.032 to 0.350)	0.019	0.172 (−0.023 to 0.367)	0.083
Baseline SBP	−0.486 (−0.661 to −0.310)	<0.001	−0.461 (−0.643 to −0.279)	<0.001
Trough bisoprolol concentration	−0.490 (−1.691 to 0.712)	0.420	−0.055 (−1.209 to 1.098)	0.924
Peak bisoprolol concentration at week 6	−0.201 (−0.343 to −0.060)	0.006		
Concomitant with amlodipine	1.632 (−3.877 to 7.140)	0.558	−1.897 (−7.065 to 3.271)	0.468
**Change in clinic DBP**				
Sex	2.257 (−0.234 to 4.749)	0.075	2.243 (−0.651 to 5.138)	0.127
Age	0.066 (−0.057 to 0.188)	0.289	0.031 (−0.122 to 0.183)	0.688
Body weight	0.046 (−0.045 to 0.137)	0.103	0.023 (−0.099 to 0.144)	0.714
Baseline DBP	−0.154 (−0.278 to −0.22)	0.023	−0.170 (−0.322 to −0.018)	0.029
Trough bisoprolol concentration	−0.377 (−1.046 to 0.292)	0.266	−0.338 (−1.052 to 0.375)	0.348
Peak bisoprolol concentration at week 6	−0.232 (−0.397 to −0.066)	0.007		
Concomitant with amlodipine	0.061 (−3.022 to 3.144)	0.969	−0.784 (−3.967 to 2.399)	0.626
**Change in clinic HR**				
Sex	−0.700 (−3.681 to 2.281)	0.642	−1.539 (−4.408 to 1.329)	0.289
Age	−0.086 (−0.230 to 0.057)	0.236	−0.192 (−0.322 to −0.052)	0.008
Body weight	0.009 (−0.098 to 0.116)	0.864	−0.031 (−0.152 to 0.090)	0.617
Baseline HR	−0.348 (−0.473 to −0.222)	<0.001	−0.403 (−0.528 to −0.279)	<0.001
Trough bisoprolol concentration	−0.588 (−1.367 to 0.191)	0.137	−0.520 (−1.232 to 0.193)	0.151
Peak bisoprolol concentration at week 6	−0.080 (−0.205 to 0.046)	0.207		
Concomitant with amlodipine	2.097 (−1.492 to 5.685)	0.249	1.946 (−1.235 to 5.126)	0.227

*SBP, systolic blood pressure; DBP, diastolic blood pressure; HR, heart rate; CI, Confidence Interval*.

## Discussion

Bisoprolol is moderately lipophilic with a volume of distribution of about 3.5 L/kg and its plasma protein binding is ~30% (34, 35). It has been reported previously that the oral clearance of bisoprolol correlated with body weight and GFR (12). We found significant associations of bisoprolol plasma concentrations with body weight but not with eGFR, possibly because all the subjects had normal renal function. We found a mean increase in the peak bisoprolol plasma concentrations of 13.6% from the first dose to the dose at 6 weeks, comparable with the reported accumulation factor of 1.1–1.3 (34, 35).

Bisoprolol is metabolized by *CYP3A4* and to a lesser extent by *CYP2D6* (36), but we did not find any significant effect of the *CYP2D6* metabolizer groups and *CYP3A5* polymorphisms examined on the peak and trough plasma concentrations of bisoprolol. Previous studies have generally found no effect of *CYP2D6* polymorphisms on bisoprolol pharmacokinetics (12, 23, 37, 38), but one study reported there were effects of CYP2D6^*^4 on the dose of bisoprolol used (39) and another reported effects of CYP2D6^*^2A on the plasma concentration and BP response (40). Overall, there is no evidence that the CYP2D6^*^10 polymorphism, which is common in East Asians (41) has any effect on bisoprolol pharmacokinetics, in contrast to propranolol and metoprolol pharmacokinetics, which are highly influenced by the CYP2D6^*^10 polymorphism (42, 43). To our knowledge there is no previous study on the effects of the CYP3A5^*^3 polymorphism or any polymorphism in *CYP3A4* on bisoprolol pharmacokinetics and pharmacodynamics.

The reductions in BP and HR were related to the baseline values for SBP and HR but there was no association with bisoprolol plasma concentrations or the polymorphisms in *CYP2D6* and *CYP3A5* examined. There are conflicting reports on the effects of *CYP2D6* genotype on the hemodynamic and clinical responses to metoprolol but recent meta-analyses found there was a significant effect on HR and BP responses corresponding with the marked effect on pharmacokinetics. The Dutch Pharmacogenomic Working Group recommended screening the *CYP2D6* genotype when metoprolol is prescribed (44). However, no effect of the *CYP2D6* genotype on the BP response to bisoprolol has been found previously (45). and genetic variants in the pharmacodynamic pathways such as the β1-adrenoceptor (*ADRB1*) may be more useful in predicting the response to β-blockers in general (46).

The reduction in clinic BP of 14.3 ± 10.9/8.4 ± 6.2 mmHg with bisoprolol 2.5 mg daily is greater than that reported with 5 mg daily (10.2/8.0 mmHg) in the bisoprolol prescribing information (34, 35). There was a placebo run-in but no parallel placebo group in our study so some of the BP change may be a placebo effect, although the changes in ABP were similar to the clinic BPs and ABP is less influenced by placebo effects. It is known from empirical observation that Chinese patients are more sensitive to propranolol than Caucasians and smaller doses are generally used. Zhou et al. (47) showed that Chinese men had greater sensitivity than white men to the effects of propranolol on HR and BP based on the responses in relation to plasma concentrations of the drug. The authors concluded that the increased sensitivity may have been partly related to decreased protein binding of propranolol, but considered that other factors must be involved. The clearance of propranolol was significantly greater in the Chinese subjects compared to the white group in that study, although it would be expected that Caucasians would have greater clearance of propranolol than Chinese subjects overall based on the high frequency of CYP2D6^*^10 IMs in Chinese causing reduced propranolol clearance (43). It is not known if Chinese subjects are more sensitive than Caucasians to other β-blockers but there are no obvious differences in the frequency of genetic variants in the pharmacodynamic pathways such as the G protein-coupled receptor kinase 4 (*GRK4*) variants, which may be related to reduced sensitivity to β-blockers in people of African origin (48).

This study had several important limitations. The single blood samples taken for peak levels were all taken at 3 h post dose but these will have missed the true peak levels in many patients which are reported to occur at a median of 3–4 h post dose. We only examined the polymorphisms in *CYP2D6* that are common in Hong Kong Chinese patients and we did not test for rare variants or for gene duplications or tandem repeats which are relatively common in this population (19), so the *CYP2D6* activity score may not be accurate. There were few PMs among these subjects and only one subject with no functional *CYP2D6* alleles so we cannot be certain about the effect of total lack of *CYP2D6* activity on bisoprolol pharmacokinetics. Likewise, the CYP3A5^*^3 polymorphism does not predict total CYP3A activity and there may be an advantage to assess the effect of *CYP3A* combined genotypes (49). However, the reduced function CYP3A4^*^22 (*rs35599367*) variant is usually absent in East Asians whereas the CYP3A4^*^1G (*rs2242480*) variant is common with an allele frequency of about 27% but its function is uncertain (50). We did not analyze the CYP3A4^*^1G variant in this study so we cannot exclude an effect of *CYP3A* combined genotypes on bisoprolol pharmacokinetics. Lastly, the number of subjects in the study is relatively small so we cannot exclude a small effect of these genotypes.

## Conclusion

There was no significant effect of the common polymorphisms in *CYP2D6* and the CYP3A5^*^3 polymorphism on the peak and trough plasma concentrations of bisoprolol or the BP and HR responses after 6 weeks treatment with bisoprolol 2.5 mg daily in Chinese hypertensive patients in this study. Genotyping for these variants would appear to have no benefit in predicting the hemodynamic response to bisoprolol in this population.

## Data Availability Statement

The raw data supporting the conclusions of this article will be made available by the authors, without undue reservation.

## Ethics Statement

The study involving human participants were reviewed and approved by the Joint Clinical Research Ethics Committee of the Chinese University of Hong Kong and New Territories East Cluster (CUHK-NTEC). The study was performed in accordance with the ethical standards laid down in the Declaration of Helsinki and subsequent revisions. All patients signed the Informed Consent.

## Author Contributions

SC and WZ analyzed the data and wrote this manuscript. BT and TC designed the research project. TC and WZ included the patients and followed this study. WZ and CH performed the experiments. BT, AK, and CH revised this manuscript. All authors contributed to the article and approved the submitted version.

## Conflict of Interest

BT has acted as consultant or speaker for Merck Serono for which he received honoraria. The remaining authors declare that the research was conducted in the absence of any commercial or financial relationships that could be construed as a potential conflict of interest.

## Publisher's Note

All claims expressed in this article are solely those of the authors and do not necessarily represent those of their affiliated organizations, or those of the publisher, the editors and the reviewers. Any product that may be evaluated in this article, or claim that may be made by its manufacturer, is not guaranteed or endorsed by the publisher.
